# On the Evolution of the Mammalian Brain

**DOI:** 10.3389/fnsys.2016.00031

**Published:** 2016-04-19

**Authors:** John S. Torday, William B. Miller

**Affiliations:** ^1^Evolutionary Medicine Program, University of California- Los Angeles, Los Angeles, CA, USA; ^2^Independent ResearcherParadise Valley, AZ, USA

**Keywords:** evolution, brain, entropy, lipids, endothermy, skin-brain, exaptation, self-organization

## Abstract

Hobson and Friston have hypothesized that the brain must actively dissipate heat in order to process information (Hobson et al., 2014). This physiologic trait is functionally homologous with the first instantation of life formed by lipids suspended in water forming micelles- allowing the reduction in entropy (heat dissipation). This circumvents the Second Law of Thermodynamics permitting the transfer of information between living entities, enabling them to perpetually glean information from the environment, that is felt by many to correspond to evolution *per se*. The next evolutionary milestone was the advent of cholesterol, embedded in the cell membranes of primordial eukaryotes, facilitating metabolism, oxygenation and locomotion, the triadic basis for vertebrate evolution. Lipids were key to homeostatic regulation of calcium, forming calcium channels. Cell membrane cholesterol also fostered metazoan evolution by forming lipid rafts for receptor-mediated cell-cell signaling, the origin of the endocrine system. The eukaryotic cell membrane exapted to all complex physiologic traits, including the lung and brain, which are molecularly homologous through the function of neuregulin, mediating both lung development and myelinization of neurons. That cooption later exapted as endothermy during the water-land transition (Torday, 2015a), perhaps being the functional homolog for brain heat dissipation and conscious/mindful information processing. The skin and brain similarly share molecular homologies through the “skin-brain” hypothesis, giving insight to the cellular-molecular “arc” of consciousness from its unicellular origins to integrated physiology. This perspective on the evolution of the central nervous system clarifies self-organization, reconciling thermodynamic and informational definitions of the underlying biophysical mechanisms, thereby elucidating relations between the predictive capabilities of the brain and self-organizational processes.

## Introduction

The origins of consciousness and the evolution of physiologic pathways in mammalian brain are arguably among the most challenging of all evolutionary puzzles. It is becoming increasingly evident that the proper point of initiation of any understanding of these phenomena channels through a fuller understanding of the capacities of individual and networked cells and even more particularly, a reassessment of the significance of the unicellular zygotic phase of all eukaryotes (Minami et al., 2007; Ikeda et al., 2010). If considered from within that cellular frame, the development of any higher level of consciousness must relate to an enabling continuum of physiologic evolution that begins from that unicellular form through which all sentient eukaryotes must recapitulate. Furthermore, such a path would necessarily extend beyond any prior assumptions of multicellular physiological development as a simple progression forward from unicellular life. Instead, eukaryotic physiology must be evaluated through the unfamiliar perspective that all the consequential processes that have antecedents from within the unicellular form retain a consistent and inherent anchor within that origin throughout development. Further too, those same initiating factors remain foundational throughout organic development not only at the level of any individual organism, but also throughout any evolutionary narrative.

This perspective is underscored by two significant biological principles. First, life is cognition at every scope and scale (Baluška and Mancuso, 2009; Shapiro, 2011; Miller, 2013; Lyon, 2015). And second, and conditional upon the first, all complex physiologic traits have evolved from the unicellular state as derivative exaptations of the complex cellular cytoskeletal elements and cell membrane as well as the crucial genetic material that is generally accredited as the centrality of that process (Torday, 2013, 2015a). All of these cellular constituents consistently inter-react to create any functioning cell. And since all cells have cognitive capacity, then, physiology is best understood as both a continuous enactment of cellular “self” and the biologic means by which that cellular “self” is maintained and advanced within cellular boundaries throughout all eukaryotic biology.

If the reality that life depends upon cognition is embraced, then in turn, any such cognition is naturally a property that must have devolved from a pre-existing physical state and the conditions that preceded that faculty. Therefore, life must adhere to basic physics, and then too, cognition must as well. Accumulating evidence supports that an appropriate frame for understanding the evolution of cognition lies within an inferential understanding of physics in a quantum informational framework as expressed in biological terms. Indeed, recent research is demonstrating that quantum processes are essential to life (Aerts et al., 2011; Wang et al., 2013). Awareness is both content and the awareness of that content which then, through quantum inference, participates in the settling of cognitive ambiguities (Conte et al., 2009). It has been proposed that many elements of the cytoskelton are crucial to the process of consciousness, particularly microtubules that demonstrate coordinated vibrational beat frequencies that may produce quantum coherences that permit the collapse of the superimposition of possibilities inherent to quantum phenomena (Hameroff and Penrose, 2014). Additional research has implicated other intracellular participants in information transfer and cognition in partnership with microtubules, such as actin filaments and collagen (Friesen et al., 2015). Similar types of quantum signal propagation have been observed within tubulin subunit proteins that comprise microtubules, particularly in the chromophores in light harvesting photosynthetic complexes (Craddock et al., 2014). Similar quantum phenomena have also been ascribed to non-polar protein interiors and membrane lipid peroxidation processes that interact either directly with microtubules or indirectly through serotonin production (Tonello et al., 2015).

With that as precursor, it can be maintained that in direct terms, any system of cognition that might eventually be embodied in the mammalian brain must be based upon quantum processes that are similar to those that produce an exchange of information between elemental receptive entities (Nurse, 2008; Walker and Davies, 2013). It is a necessary conclusion then, that physiology supports this preconditioning state in eukaryotes (Torday, 2015a). However, on a thermodynamic basis, this necessary exchange of information is also a transfer of energy linked to heat production. Therefore, any cognitive action as a form of cellular coherence can be better understood as both an information exchange and reciprocally then, as energy conversion and transfer (Adolphs and Renger, 2006; Dahlberg et al., 2015).

When this is our consideration, then “self” is best considered as a function of both energy and information transfer whose targets need not be identical. Since these latter two faculties are amply demonstrated within unicellular life, then “elf” is also invested at that scale. Even within that scope, when communication is accomplished, and information is transmitted as communication between a sender and a receiver, then both independent entities are now linked through that process (De Loof, 2015). It would seem evident then that self-awareness arises as a derivative of physical processes, based upon coordinate and reicprocating functions that understand its context within the larger organism. In multicellular organisms then, self-awareness becomes a function of cellular constituencies that both compete and collaborate.

Therefore, in a cellular frame, self-awareness as a cognitive function must be dependent upon the discrimination of cellular status through active cell homeostasis as the basis upon which physiology is constructed (Takada and Jameson, 2009). That faculty of discrimination of biologic status as opposed to the external environment as assessed through differential cellular physiological function thereby becomes the basis for self-awareness. “Self” is therefore cognitive awareness of homeostatic flux as maintained within cellular boundaries. Since homeostatic flux is itself dependent upon continuous physiologic activity, then “self” even as its own property must then be interpreted through physiological mechanisms.

Within any quantum biologic frame, dissipation of energy as generated heat can be viewed in terms of disappearance and emergence of coherence within and between cells (Engel et al., 2007; Larson, 2014). This parallel process is best understood as based upon cellular properties effectuated in support of the imperative of cellular homeostasis to maintain self. Such coherence can properly be construed as the ability of biologic organisms to resolve ambiguities toward survival in preferred states. Yet, in biologically active systems, all such actions have a thermodynamic (energy) cost, producing the need for active heat exchange with the environment.

In biological terms, this thermodynamic gradient is enacted as a series of downhill thermodynamic paths balancing energy usage and output with energy (heat) dissipation (Aledo and del Valle, 2004). It can be presumed then that this is best achieved in the multicellular form. Therefore, multicellular physiology is a mechanistic solution for the utilization of energy and heat dissipation in order to control local homeostasis upon which “self” is dependent. It is this process in series that yields multicellular entities directed toward that same end that are appraised by us in biologic terms as evidence of “self-organization.”

Any such reiterative process must always represent a continuum from basic thermodynamic principles brought forward as varied biologic manifestations based upon energy utilization and information transfer. Although there are crucial transitions within biologic phenomena by which free energy in thermodynamic terms must be differentiated from variational minimal free energy in biologic information space, the dissipation of heat inherent to all life forms can be considered as active suppression of free energy toward its minimum, a process that has been directly linked to how the brain acts to limit prediction errors (Friston et al., 2006). In this manner, any agent tends toward self-organization by minimizing free energy and thereby lowering any probablility of surprise. It proceeds in that direction through reciprocating interaction with its environment that rests upon Bayesian inferences about its context (Friston, 2009). In a cellular-based dynamic for cognition such as is being proposed, each cell is recognized as a discrete cognitive entity that acts both individually and collectively. Therefore, the statistical power of Markov blankets is pertinent as a descriptor of the manner in which cellular membranes uphold their intracellular matrix and separate from extracellular influences. Within the general conditions of that frame, Bayesian inferences are based upon random dynamical systems as features of variational free energy and local coupling (Friston et al., 2014). However, in a system based upon self-referential cognition as a thermodynamically-derived state function, there are direct biological limits placed upon the bounded dispersion of sensed states by which cells experience epiphenomena and the outward environment (Friston, 2013). Therefore, in quantum biological circumstances, these critical models can be subject to modification within the proscriptions of self-awareness and the constraining physiologic processes that are enacted to sustain it. The inevitability of self-organization as a form of active Bayesian inference within the spatial boundary conditions of any living organism therefore remains valid but becomes empowered. Biological uncertainties are resolved through reciprocal biological signaling in which inputs are not necessarily random, and via coupling in quantum systems that are subject to both local and non-local correlations (Al-Khalili and McFadden, 2014). Therefore, the ability of biological organisms to settle ambiguities within expanded inferential terms extends beyond typical statistical informational matrices, and thereby becomes a definitional crux of biological action. In multicellular entities then, any instrinsic drive toward a mandate to minimize variational free energy places each cell not merely as “in” its environment, but as a reciprocating organic entity that is “of” its external milieu at the same time. In this way, it becomes a specific embodiment of the “good regulator system” (Conant and Ross Ashby, 1970). It is continually isomorphic through self-assessment of its internal milieu as opposed to its external environment, of which it is a both quantum observer and participant. This consistent reciprocation becomes the specific basis for its homeostatic regulatory mechanism, both minimizing variational free energy, and underscoring its self-referential appraisal of conditional status.

Importantly, the “self” that exists within all living entities is itself a state function. Consciousness as awareness of an external environment is present in every form of life that is differentiated from the inanimate (Giuditta, 2010; Trewavas and Baluška, 2011). As such, it must be considered as a basic property of biology as a quantum system. Similar to energy and enthalpy, that state function may have differing values but is independent of the exact number of steps that were required to create its exact moment. Therefore, entropy, enthalpy, and self are linked, yet separable variables as state functions that are all directed toward maintaining homeostatic balance against environmental stresses. Although homeostatic status is dependent upon physiologic processes to efficiently utilize energy and dissipate heat, this process still must be co-aligned with those major state functions. Therefore, it can properly be imputed that physiologic pathways maintain cellular homeostasis as the means by which biologic substrates are utilized and purposed to sustain “self,” as a basic property of living systems. Therefore, physiology becomes more than a series of interrelated processes but represents a further enactment of “self” as a state function as it is achieved through multicellular networks through achievable thermodynamic states. Physiology can then be viewed as more than a means of maintaining protective cellular homeostasis, but further, as an active agency that both permits and sustains “self.” This is accomplished through cell-cell communication as the exchange of information that underscores self-identity. This activity is an energy-intensive process. Multicellular organisms might then be assumed to represent that form that best utilizes energy transformation for information sharing, and thereby, self-organization can now be best understood in a biologic context as a series of linked processes by which thermodynamically advantaged solutions to environmental stresses are achieved through form.

Although not obvious, multicellularity need not have been a necessary evolutionary outcome. Intracellular engineering might have led to enormously large, efficient and capable single cells. There is an analogy in the viral realm to support this case. The giant mimiviruses are larger in size than some bacteria, and have larger genomes (Moreira and Brochier-Armanet, 2008; Raoult and Forterre, 2008). However, in the cellular realm, this has not been our known biologic outcome. A salient question within biology might be “why didn't evolution lead to single extremely large and efficient cells as the dominant biologic players?”

That answer lies within physiologic mechanisms that extend forward from unicellular roots. These are based upon stable principles of evolutionary development that can be traced from those unicellular origins as thermodynamically effective outcomes for the maintenance of cellular identities and cellular homeostasis directed by a sustaining “self.”

So we know where consciousness/mind originated from, and how it appears in its fully formed state, but how did it transition from paramecia to Einstein? The key to such an analytic approach to the evolution of mind through paths of complex physiology is the realization that any given physiologic trait is the permutations and combinations of pre-existing unicellular mechanisms and physiologic traits (Torday, 2015b). This does not proceed in a linear, arithmetic fashion, but as Boolean contingencies on previous events in the history of the organism. Knowing the molecular “parts list” and the contexts in which they have existed in previous iterations and persist as current physiology and metabolism provides important clues to how and why they are relevant to brain evolution.

One important contextual clue is the water-land transition that occurred some 300 million years ago due to the CO_2_ “greenhouse effect” (Ward et al., 2006), drying up lakes, rivers, and ponds (Romer, 1949). That precipitated several specific gene duplications in vertebrates due to the existential stress of having to adapt to land (Torday, 2013), providing insights to pivotal physiologic changes in vertebrate physiology- the lung, kidney, skeleton, skin. This paper is predicated on the hypothesis that like those visceral organs, the brain also evolved under selection pressure, as first proposed by Hughlings-Jackson (Franz and Gillett, 2011), and reinforced by Gottlieb (2007), who pointed out the norms of reaction in brain structure/function. Thereby, it can be advanced that there is an underlying cellular apparatus that appraises self through boundaries and proscriptions, thereby purposing “self” toward common cellular solutions to maintain homeostasis in reaction to environmental stresses that affect all the cellular constituencies of macro-organisms. This is achieved through physiological/metabolic pathways.

## Genetic changes associated with the water-land transition facilitate vertebrate evolution

The initiating factor for vertebrate evolution was the insertion of cholesterol into the phospholipid bilayer (Miao et al., 2002), rendering the membrane more compliant by thinning it out and making it more permeable for gas exchange (Torday and Rehan, 2012). This is the fundament of vertebrate evolution that enabled endo/exocytosis, increased metabolism, and facilitated locomotion (Perry and Carrier, 2006).

The beginnings of the evolution of the visceral organs in vertebrates is the adaptation of water-based life forms to a primary existence on land. In support of this, there were two gene duplications for the Parathyroid Hormone-related Protein Receptor (PTHrPR) (Pinheiro et al., 2012) and the Beta Adrenergic Receptor (βAR) (Aris-Brosou et al., 2009), accompanied by two gene mutations, for the Glucocorticoid Receptor (GR) (Bridgham et al., 2006) and type IV collagen (MacDonald et al., 2006). The first three of these genetic changes were critically important in the evolution of land adaptive visceral organ changes. The best known is the PTHrP signaling mechanism, which is necessary for the formation of lung alveoli (Rubin et al., 1994), the primary mechanism for lung evolution (Torday and Rehan, 2007)—deletion of PTHrP results in failed alveolar formation (Rubin et al., 1994). It also affects bone (Karaplis and Goltzman, 2000), skin (Wysolmerski et al., 1998), kidney (Hochane et al., 2013), and brain (Liu et al., 2013), though not as profoundly as the lung- mice lacking the PTHrP gene die at birth of pulmonary insufficiency (Karaplis et al., 1994). The βAR was necessary for the earlier stages of lung evolution, increased βAR density in the pulmonary microcirculation allowing for blood pressure regulation independent of the systemic circulation (West and Mathieu-Costello, 1999). This adaptation was critical for the increase in gas exchange surface area of the lung, without which, every time there was a physiologic reaction to stress the microcirculation of the nascent lung would have been damaged. The GR evolved from the Mineralocorticoid Receptor (MR) (Bridgham et al., 2006), likely due to the elevated blood pressure on land vs. water (Volkmann and Baluska, 2006). The addition of two amino acids to the MR resulted in the evolution of the GR (Bridgham et al., 2006); the other positive selection for the GR during the water-land transition was its molecular induction by activation of βARs (Maier et al., 1989), effectively reducing blood pressure under stress conditions. The type IV collagen mutation that causes Goodpasture's Syndrome was adaptive for both the alveolus and glomerulus because it is hydrophobic (MacDonald et al., 2006), forming a barrier against the loss of fluid and electrolytes from the lung and kidney on land. However, people expressing this isomer of type IV collagen can develop autoantibodies to it, inhibiting gas exchange in the alveolus and blood filtration in the glomerulus, eventually resulting in death (Greco et al., 2015).

There is an interesting fundamental mechanistic difference between the PTHrP and βAR receptor gene duplications and the GR mutation, and that of the type IV collagen matrix protein, all of which were responses to physiologic stress caused by increased blood pressure, generating oxygen radicals in the microcirculation (De Nigris et al., 2001). In the case of the receptors, their expression was constrained by their previously evolved down-stream signaling pathways (Jordan et al., 2000), referred to in conventional evolutionary biology as terminal addition (Jacobs et al., 2005), whereas the type IV collagen mutation had no such specific servo-regulatory constraints, explaining why the consequent disease.

## On the evolution of endothermy

Given the described step-wise empiric adaptation to land, there were undoubtedly stages in this process at which the lung was inefficient for gas exchange, resulting in hypoxia, the most potent physiologic stressor known. Such stress conditions would have resulted in spates of catecholamine production by the adrenal gland, alleviating the constraint of an inefficient lung by stimulating surfactant production by the alveoli (Lawson et al., 1978), acutely increasing alveolar distensibility and thus oxygenation. Over time, such episodes of over-distension of the alveoli would have culminated in the formation of additional alveoli, PTHrP acting to form more alveolar units (Rubin et al., 1994), accommodating oxygen deficiency constitutively. In tandem, catecholamines would have stimulated fatty acid secretion by fat cells in the periphery (Lawson et al., 1978), increasing metabolism and body heat (Lee et al., 2015). Again, over time this mechanism would have given rise to a constitutive increase in body temperature, or endothermy. As evidence for this mechanism of evolution, PTHrP signaling appears in the pituitaries of mammals (Mamillapalli and Wysolmerski, 2010) and birds (Nakayama et al., 2011), and stimulates corticosteroid production by the adrenal cortex (Mazzocchi et al., 2001) in association with increased microvascularization of the adrenal medulla (Wurtman, 2002). Consequently, in the pituitary PTHrP amplifies ACTH (Mamillapalli and Wysolmerski, 2010), and in the adrenal cortex it stimulates corticoid production (Kawashima et al., 2005). The associated increase in angiogenesis within the adrenal medulla amplifies corticosteroid stimulation of the rate-limiting step in catecholamine synthesis, Catecholamine-O-Methyltransferase (Nic a' Bháird et al., 1990). The enhanced microcirculation within the medulla was likely due to the increased production of PTHrP within the adrenal cortex passing through the adrenal medulla since PTHrP is angiogenic (Isowa et al., 2010). The other consequence of increased catecholamine production elevating body temperature was the evolution of lung surfactant in adaptation to endothermy (Torday, 2015a). The composition of the surfactant phospholipid changes to dipalmitoylphosphatidylcholine (Suri et al., 2012), which has a phase transition temperature of 41°C, rendering it 3-times more active than it is at 25°C (Lau and Keough, 1981). In support of this hypothetical interrelationship, catecholamines have an adaptive effect on peripheral cellular oxygenation, increasing the amount of unsaturated phospholipid in the cell membrane, making it more gas permeable (Ward et al., 2006). In contrast to this, under hibernation conditions when oxygen utilization and stress are at a minimum, there is decreased unsaturated phospholipid in the peripheral cell membranes (Lau and Keough, 1981), decreasing cellular oxygen uptake, and lung surfactant phospholipid composition reverts to its cold-blooded composition (Suri et al., 2013). Experimental support for this integrated mechanism comes from study of MAP turtles reared at different ambient temperatures, altering the composition of their lung surfactant consistent with the previously described evolutionary changes (Lau and Keough, 1981).

## On the evolution of the brain in endotherms

The evolution of endothermy/homeothermy is a milestone in vertebrate evolution shared by mammals and birds (Grigg et al., 2004). What else do these organisms share in common that might give insight to evolution? It may be of evolutionary relevance that mammals and birds are both bipedal. That trait may have been contingent on the evolution of endothermy since being warm blooded rendered metabolism much more efficient. Cold-blooded poikilotherms require multiple isoforms of the same metabolic enzyme to function efficiently at different ambient temperatures (Duarte et al., 2007). Bipedalism requires much more energy than being quadrupedal (Rodman and McHenry, 1980). Another trait held in common by mammals and birds that may be a consequence of endothermy and bipedalism is the freeing of the forelimbs for such adaptations as flight in birds, and manual manipulation in Man.

There are clues within this narrative toward the evolution of the central nervous system in the context of the convergence of thermodynamic, self-organizational and informational characteristics. Importantly, there are exaptive traits that fostered the higher consciousness of mammals and birds. The role of thermoregulation has been alluded to by Hobson et al. (2014), invoking the need to cool the brain during Rapid Eye Movement sleep. As for the self-organizational aspect, the evolution of lipid metabolism converged in endothermy and pulmonary alveolar respiration (Torday, 2015a), perhaps acting as positive selection for neuregulin, an intermediary in the Epidermal Growth Factor signaling pathway that mediates both alveolar and neuronal lipid utility. In the alveolus, neuregulin promotes surfactant phospholipid synthesis (Fiaturi et al., 2014), whereas in the brain it is the only known mechanism that mediates myelinization of axons by Schwann Cells (Li, 2015). As previously described, the co-evolution of the pulmonary and neuroendocrine systems via PTHrP signaling is more evident, and may have been the forerunner of the neuregulin exaptation. Neuregulin mediates Schwann cell myelinization of neurons in the skin (McKenzie et al., 2006), the latter bearing a strong homology with the lung and brain. Neuregulin' role in myelinization is consistent with the informational phenotype proffered by Shannon's Communication Theory (Shannon and Weaver, 1949). And the evolution of the forelimb reinforced such co-evolved mechanisms at multiple levels, referring all the way back to the advent of cholesterol in the cell membrane of unicellular eukaryotes promoting locomotion, respiration and metabolism (Perry and Carrier, 2006).

Another co-evolved molecular mechanism common to the lung, adipose tissue and brain is leptin, which is secreted by both fat cells (Adamczak and Wiecek, 2013) and the lipofibroblasts of the alveolar wall (Torday et al., 2002). The role of leptin in endothermy/homeothermy has been demonstrated by the experimental treatment of cold-blooded Fence Lizards with leptin, increasing their basal metabolic rate and body temperature (Niewiarowski et al., 2000). In the brain, leptin stimulates the arborization of the neurons (Moult and Harvey, 2008), increasing informational processing properties in the brain.

Thus, the arc of vertebrate physiologic evolution is better understood as a continuum emanating from the water-land transition (Torday, 2013, 2015a), through only a few crucial pleiotropic gene duplications and mutations that occurred in that era—the Parathyroid Hormone-related Protein (PTHrP) Receptor, the βAdrenergic Receptor (βAR), and the Glucocorticoid Receptor (GR). The PTHrPR in particular fostered the evolution of a number of key terrestrial adaptations, most importantly the lung alveolus. The βAR was similarly critical in adaptation to air breathing since its regulation of blood pressure in both the systemic and pulmonary circulations was a constraint on the expansion of the surface area of the gas exchanger (West and Mathieu-Costello, 1999).

The evolution of the vertebrate lung from the fish swim bladder (Zheng et al., 2011) was principally due to the progressive reduction in the air space formed initially by the swim bladder of fish, resulting in the increase in the gas exchange surface area-to-vascular blood supply ratio (Torday and Rehan, 2012). In order for that to occur, the alveolar epithelial type II cells lining the air space/alveolus had to increase the efficiency of surfactant surface tension reducing capacity to counter the increasing surface tension due to the reduction in the alveolar diameter- the surface tension of a sphere being inversely proportional to its diameter (Law of Laplace). It is this dynamic interplay of epithelial-mesenchymal interactions mediated by soluble paracrine growth factors and their cognate receptors (Torday and Rehan, 2012) that orchestrated the evolution of air breathing, from the swim bladder of fish to the lungs of amphibians, reptiles, mammals and birds.

It is through understanding these sorts of biophysical mechanisms that the evolution of the brain, particularly as it relates to self-organizational processes, can be explored as a productive reconciliation between thermodynamic necessities and informational requirements. At each moment, organizational problems secondary to system wide epiphenomena are being solved. It has been hypothesized that the interaction between the evolving lung and neuroendocrine system gave rise to endothermy in a series of steps. Intermittent periods of hypoxia during the water-land transition would have caused physiologic stress, hypoxia being the most potent physiologic stressor known in vertebrates. The stress would have been alleviated by the production of catecholamines by the adrenal medulla, relieving the initial constraint on gas exchange by stimulating the alveolar secretion of surfactant, rendering the evolving alveoli more distensible, acutely increasing their surface area for gas exchange. Over the long haul, these bouts with hypoxia would have fostered more alveoli since the distension of the alveoli stimulates PTHrP signaling, fostering more alveoli. In tandem with the stimulation of lung evolution, catecholamines would have stimulated the secretion of fatty acids from fat cells, in turn leading to increased metabolism and body temperature. Consequently, endothermy evolves over time in adaptation to terrestrial life, as a thermodynamically efficient solution to the chain of informational transfer that supports life and upon which “self” is based. In support of this hypothesis, PTHrP signaling for ACTH appears in the mammalian and avian pituitaries and in the adrenal cortex in association with increased microvasculature in the adrenal medulla. These physiologic changes would have enhanced catecholamine production in response to stress, synergizing the evolution of both the alveoli and endothermy. Systematically, the physiologic effect of catecholamines on the gas permeability of the cell membrane promotes oxygenation- the catecholamines promote the population of the cell membrane by unsaturated phospholipids, rendering it more fluid and therefore more permeable to gas exchange. This property is likely causal since the opposite effect is seen during hibernation and torpor, regarding both the composition of the cell membrane phospholipids, and that of the lung surfactant.

Therefore, factors that seem separable on an evolutionary basis are in fact united. They extend through demonstrable evolutionary paths that must remain adherent to those basic principles that underscore all such connections. This then provides for neuregulin mediation of both lung development and axonal myelinization, or the skin-brain connection, with their joint embryological connections (Foster, 2012). The Friston-Hobson (Hobson et al., 2014) theory of brain cooling coalesces with endothermy as both become implicit with regard to physical requirements in support of information systems. Therefore, the suspension of thermoregulation during Rapid Eye Movement sleep infers a “reverse evolution” reflecting that endothermy evolved from poikilothermy, and “Brain Cooling” becomes functionally homologous with the implementation of catecholamines for thermoregulation within the brain.

Complex physiology emerged as exaptations from the unicellular realm (Torday, 2015a). Therefore, self-organization can be understood as a direct means of protecting cellular physiological homeostasis that in turn sustains self-identity within consonant thermodynamic roots. At every scope and scale, self-aware cells enact solutions to environmental stresses according to thermodynamically efficient paths. “Self” exists within those thermodynamic necessities at the cellular level and then proceeds through multicellular reiterative physiological mechanisms to become, in an eventual series, our human brain. This accounts for a mammalian brain that is, in thermodynamic terms, an open system on an entropic basis and is energetically dissipative (Freeman and Vitello, 2011; Varpula et al., 2013).

Therefore, the distributive nature of mammalian cognition across widely separated cellular networks can be concluded to be derivative of both cellular-based cognition and also physiological mechanisms permitting energy dissipation in a manner that yields neurohumoral coherence across space-time. Physiological mechanisms both permit and support cellular “self” as a state function that emanates from unicellular origins and perpetually exists within that frame. That resultant network of cellular self-identity is always adherent to thermodynamic limits and is further delimited by cellular homeostasis that is itself dependent upon physiological pathways extending forward from their unicellular origins. Multicellular networks reiterate toward mammalian cognition by purposing self-organization as responsiveness to cellular self-identification, ever dependent upon the maintenance of cellular homeostatic flux boundaries, and sustained and advanced by cellular physiologic mechanisms in continual adaptation to epiphenomena.

## Author contributions

JT contributed 50%; WM contributed 50%.

## Funding

JT has been supported by NIH Grant HL055268

### Conflict of interest statement

The authors declare that the research was conducted in the absence of any commercial or financial relationships that could be construed as a potential conflict of interest. The reviewer KF and handling Editor declared a current collaboration and the handling Editor states that the process nevertheless met the standards of a fair and objective review.
